# Inferring phylogeny and speciation of *Gymnosporangium* species, and their coevolution with host plants

**DOI:** 10.1038/srep29339

**Published:** 2016-07-07

**Authors:** Peng Zhao, Fang Liu, Ying-Ming Li, Lei Cai

**Affiliations:** 1 State Key Laboratory of Mycology, Institute of Microbiology, Chinese Academy of Sciences, Beijing 100101, China

## Abstract

*Gymnosporangium* species (Pucciniaceae, Pucciniales) cause serious diseases and significant economic losses to apple cultivars. Most of the reported species are heteroecious and complete their life cycles on two different plant hosts belonging to two unrelated genera, i.e. *Juniperus* and *Malus*. However, the phylogenetic relationships among *Gymnosporangium* species and the evolutionary history of *Gymnosporangium* on its aecial and telial hosts were still undetermined. In this study, we recognized species based on rDNA sequence data by using coalescent method of generalized mixed Yule-coalescent (GMYC) and Poisson Tree Processes (PTP) models. The evolutionary relationships of *Gymnosporangium* species and their hosts were investigated by comparing the cophylogenetic analyses of *Gymnosporangium* species with *Malus* species and *Juniperus* species, respectively. The concordant results of GMYC and PTP analyses recognized 14 species including 12 known species and two undescribed species. In addition, host alternations of 10 *Gymnosporangium* species were uncovered by linking the derived sequences between their aecial and telial stages. This study revealed the evolutionary process of *Gymnosporangium* species, and clarified that the aecial hosts played more important roles than telial hosts in the speciation of *Gymnosporangium* species. Host switch, losses, duplication and failure to divergence all contributed to the speciation of *Gymnosporangium* species.

The plant genus *Malus* (Rosaceae family) comprises 33 to 55 species, and species in this genus were mainly cultivated for fruit production^1^. They have great economic importance worldwide, especially in China, where apple production accounts for more than half of world production (http://apps.fas.usda.gov/psdonline/circulars/fruit.pdf). Cedar-apple rust diseases, caused by species from genus *Gymnosporangium* R. Hedw. ex DC. (1805) (Pucciniaceae, Pucciniales), are one of the most devastative diseases occurring on apple cultivars. The *Gymnosporangium* species can infect leaves, fruits and stem of *Malus* species, and cause premature defoliation and eventually kill the plants^2^,^3^.

The genus *Gymnopsorangium* was established to accommodate *G. fuscum* DC. on *Juniperus sabina* L^4^. Up to date, around 57 species have been reported worldwide in this genus^5^, among which, 17 species were reported with their aecial stage on *Malus* species^6^. These 17 species were described by various mycologists in Asia, Europe and North America and they had heteroecious and demicyclic life cycles^7^. To complete the life cycle, these species have their aecial stage on *Malus* species, and telial stage on *Juniperus* or *Chamaecyparis* species^5^,^8^.

Taxonomic studies on *Gymnopsorangium* have long been conducted in Europe, North America and Japan^9^,^10^,^11^. Among the five spore stages (i.e., spermogonium, aecium, uredinium, telium and basidium), morphological characters in aecial and telial stages were of significant importance for species recognition^5^,^10^,^11^,^12^. However, due to lack of a consistent species concept, different taxonomic systems have employed various morphological characters, and aecial or telial host range for species delimitation. Kern^5^ presented a taxonomic monograph of *Gymnosporangium* and emphasized the importance of morphology in aecial and telial stages, the phylogenetic significance of those emphasized criteria however, have not been evaluated. In recent years, molecular data have been more and more frequently employed to resolve taxonomic issues especially for species with little morphological variation^13^,^14^. However, most of the recent molecular taxonomic studies simply recognized well supported clades as distinct species without implementing careful examination of species boundary; thus, use of coalescent approach has been recommended in order to assess the consistency of delineated species from different models^15^,^16^,^17^.

The first report of *Gymnosporangium* in China was *G. corniforme* on *J. formosana*^18^. Subsequently, described species in *Gymnosporangium* gradually increased to five^19^,^20^. Based on the aecial spore morphology, Zhuang *et al*.^21^ collectively treated the causal agents of 12 *Malus* hosts as *G. yamadae*, which were previously recognized as *G. asiaticum*, *G. fenzelianum*, *G. globosum*, *G. laeve* or *G. yamadae*^19^,^20^ based only on the morphological similarities in the aecial stage. In addition, Zhuang *et al*.^21^ speculated that *J. chinensis* was the telial host of *G. yamadae*, but lack experimental or molecular evidences.

Previously, uredinologists held the opinions that rust fungi have coevolved with their hosts over long time, and cospeciation played the most important role in host and rust fungi evolution^22^,^23^. However, recent study indicated that host jump, rather than cospeciation, were the main speciation events driving the evolution of rust fungi^24^. In the genus *Gymnosporangium*, controversial opinions were hold among researchers toward the evolutionary relationships with host species. Moran^25^ suggested that *Gymnosporangium* species are tightly coevolved with their hosts despite the absence of an overall cospeciation pattern. In contrast, based on the phylogenetic relationship of several *Gymnosporangium* species, Novick^26^ thought host switching rather than cospeciation was the primary speciation model in the group. Because only very limited *Gymnosporangium* species were included in their studies, the evolutionary history and speciation mode of *Gymnosporangium* species on *Malus* species remains largely unknown.

The objectives of the current study are: 1) to conduct the species delimitation analyses for *Gymnosporangium* species; 2) to clarify the host alternation of these recognized *Gymnosporangium* species on *Malus*; 3) to conduct the cophylogenetic analyses of *Gymnosporangium* species with their aecial host and telial host respectively; 4) to infer the speciation modes (cospeciation, duplication, host switch, loss and failure to diverge) of *Gymnosporangium* species.

## Results

### Species delimitation

**Figure 1:**
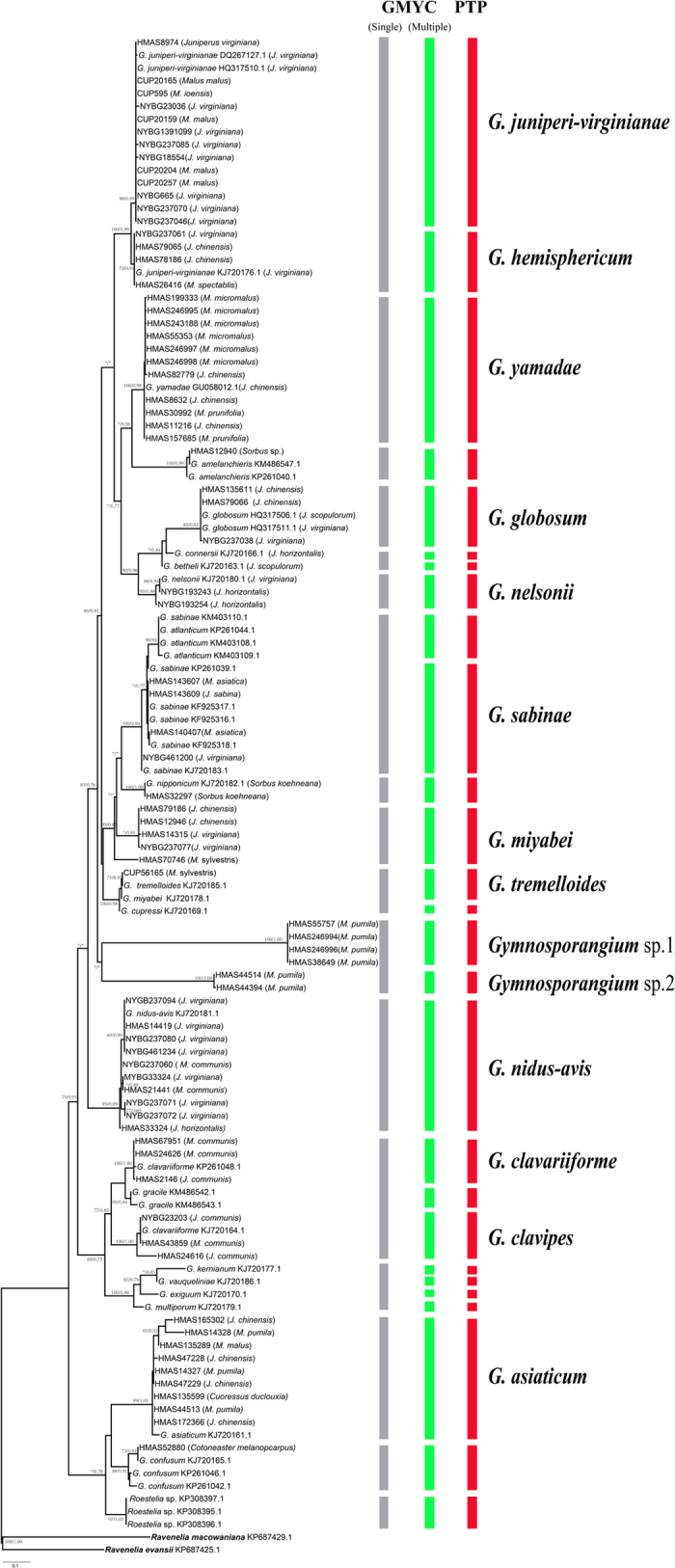
Phylogenetic trees of the combined data of the internal transcribed spacer regions and intervening 5.8S nrRNA gene (ITS) and the large subunit (LSU) rDNA obtained from parsimony analysis. Bayesian posterior probabilities (Bpp) were given immediately followed by the bootstrap values of ML on the nodes in the topology. Asterisk (*) represented bootstrap values less than 50% or Bpp less than 0.75 in the topology. The first column depicts species recognized by the single-threshold GMYC model, and second column depicts putative species recognized by multiple-threshold GMYC model. The third column depicts putative species recognized by PTP model. The name of each putative species was designated based mainly on Kern (1973).

In the GMYC analyses, totally 80 haplotypes were found from 114 specimens, and identical haplotypes and two outgroup samples were removed for final analyses. The GMYC analyses using the single- and multiple-threshold models revealed different results (Fig. 1). Both single- and multiple-threshold models were preferred over the null model of uniform branching rates. In the single-threshold analysis, confidence intervals for the estimated number of species varied from 10 to 25, and the model fitted the switch and lead to an estimation of 17 putative species. This species delimitation scenario was not well supported by the result of LR test (*P*-value = 0.3823). In the multiple-threshold analysis, the model fitted the switch in the branching patterns at 20 to 33, and the results lead to 27 putative species. In comparison with single-threshold analysis, the results from multiple-threshold analysis appeared to be more reliable because it was statistically supported by the result of LR test (*P*-value = 0.008733775^**^). In addition, due to different species delimitation scenarios based on single- and multiple-models, STEM was used to estimate the likelihood values of alternative species delimitation scenarios. Based on the protocol by Carstens & Deweya (2010), the likelihood scores of 1-species scenarios, 17-species scenarios and 27-species scenarios were analyzed. STEM analyses also supported a 27-species scenario over other species scenarios. In the PTP analyses, the ML-scenarios recognized 27 species, which was congruent with multiple-threshold GMYC models. Thus, based on concordant results from GMYC and PTP models, 27 species scenarios were recognized.

Sequences obtained from this study scattered in 14 putative species. Morphological characters from aecia, peridium, aeciospores, telia and teliospores were observed and recognized among species (Supplementary Fig. 1; Supplementary Table 1). The proper name of each putative species was determined based on these morphological characters^5^,^9^,^10^,^11^,^12^,^27^,^28^,^29^. Our results are in good agreement with the taxonomic system of Kern^5^. Two unnamed species were recognized by our molecular analyses.

### Host alternation

Based on the telial and aecial host information of specimens within each putative species, we clarified the host alternations of 10 species, which were previously unknown. The aecial and telial host ranges of each putative species were summarized in Supplementary Table 2. The telial hosts were mainly recognized in *J. chinensis*, *J. communis*, *J. horizontalis*, *J. scopulorum* and *J. virginiana*, and they did not show apparent host specificity because six morphologically distinguishable *Gymnosporangium* species shared *J. virginiana* as one of the telial hosts. In addition, *J. chinensis* was also the telial host of seven *Gymnoporangium* species. Similarly, *J. communis* was found as host of two species, i.e. *G. clavariiforme* and *G. clavipes.* In the aecial stage, *M. asiatica*, *M. micromalus*, *M. prunifolia* and *M. spectubilis* were shown to be specific to certain *Gymnoporangium* species, but *M. communis*, *M. malus*, *M. pumila* and *M. sylverstris* were shown to be the aecial hosts of two or three *Gymnosporangium* species, respectively.

### Divergent time of *Gymnosporangium* species

**Figure 2:**
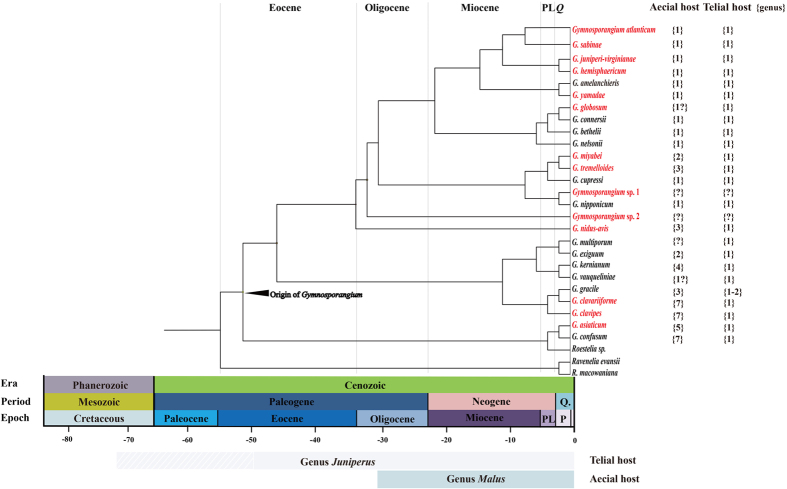
Divergence time estimation of *Gymnosporangium* species using the rDNA dataset. The chronogram was obtained from the molecular clock analysis using BEAST. The *Ravenelia* (Raveneliaceae, Pucciniales) fossil was served as a reliable calibration point to estimate the divergent time of *Gymnosporangium* species. Species with aecial host on *Malus* species were indicated in red color. The estimated divergence time of telial and aecial hosts were indicated below the geographic time scale. In addition, the potential number of telial and aecial host genera was pointed out following the name of the species based on Kern (1973) and our study.

The root of the tree was calibrated based on the age of a fossil fungus in the genus *Ravenelia*. The mean age of the node marking the split of *Gymnosporangium* species were shown in the Fig. 2. The split of *Gymnosporangium* species from the genus *Ravenelia* occurred at the Eocene epoch of the Palaeogene period in the Cenozoic era, approximately 51.7–44.3 Mya with the calibration point of genus *Ravenelia* (55.8–40.4 Mya, fossil record). It can be recognized that the aecial host ranges gradually decreased in the evolution and divergence of species in *Gymnosporangium*. The early divergent species, such as *G. asiaticum* and *G. confusum*, had relatively wider aecial host range up to seven genera^5^. However, several recently divergent species, such as *G. amelanchieris*, *G. atlanticum*, *G. bethelii*, *G. connersii*, *G. hemisphaericum*, *G. juniperi-virginianae*, *G. nelsonii*, *G. sabinae* and *G. yamadae*, had their aecial host on only one host genus. On the other hand, no clear tendency was recognized from that of the telial hosts.

### Cospeciation and cophylogeny

**Figure 3:**
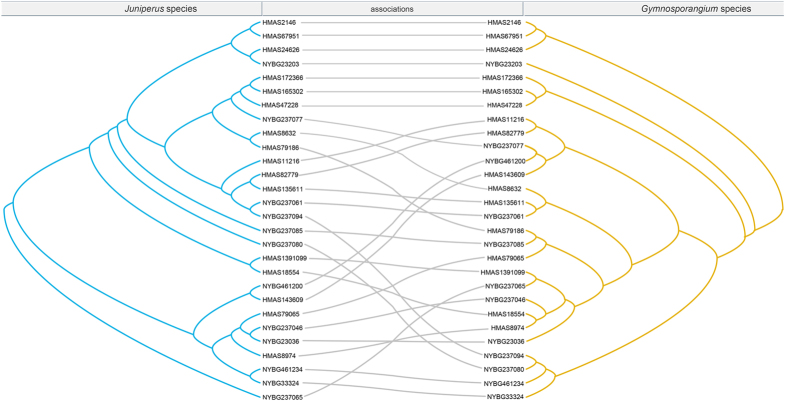
The tanglegram between specimens of *Gymnosporangium* species and telial host *Juniperus* rDNA phylogenies. Fungal (right) and host (left) phylogenies from Bayesian inference were used to generate the tanglegram using TreeMap 3.0ß.

**Figure 4:**
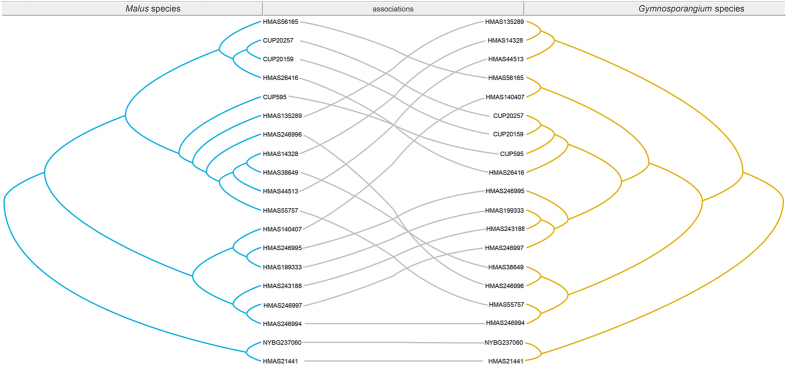
The tanglegram between specimens of *Gymnosporangium* species and aecial host *Malus* rDNA phylogenies. Fungal (right) and host (left) phylogenies from Bayesian inference were used to generate the tanglegram using TreeMap 3.0ß.

**Figure 5:**
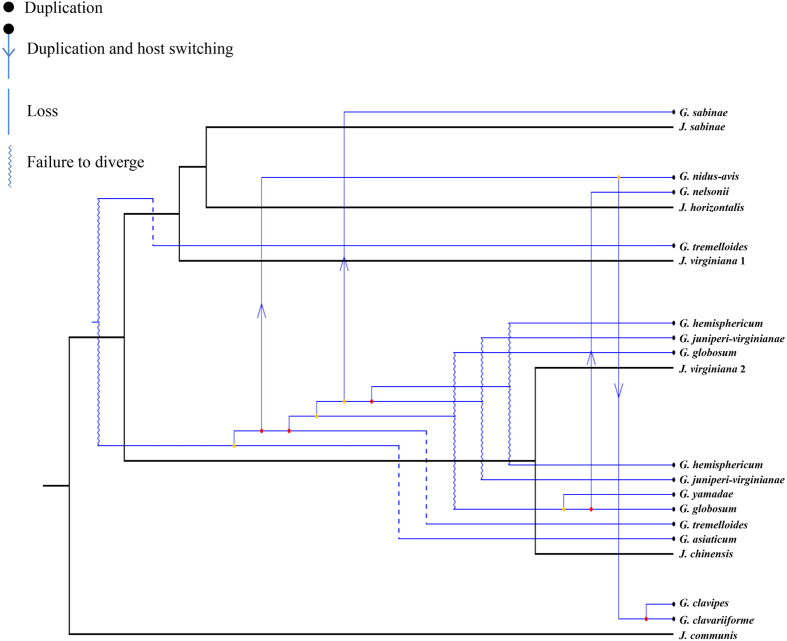
Cophylogenetic analysis of the *Gymnosporangium*–*Juniperus* pathosystem conducted with Jane 4 and using phylogenies including one representative per potential species of parasite and host. Black branches represent the host phylogeny and blue branches the parasite phylogeny. Violet lines represent original polytomies resolved by Jane to minimize the overall cost of the solution. The cost regime used for the reconstruction was by following event costs: (cospeciation = 2, duplication = 0, host switch = 1, lineage sorting = 1 and failure to diverge = 1). The best-fit reconciliation of the *Gymnosporangium* and *Juniperus* trees included 6 duplications, four host switches, three losses and finally four failures to diverge.

The tanglegrams of phylogenetic associations between *Gymnosporangium* species and their telial and aecial host species were shown respectively in Figs 3 and 4. Several codivergence events were revealed between *Gymnopsorangium* species and *Juniperus* species in Fig. 3, but global congruence between hosts and parasites was not significant. On the contrary, we found high incongruence between host and parasites. Event cost based tests by Jane 4.0 recognized the lowest overall costs recovered using the cost regimes “7”, which penalized cospeciation and assigned cost = 0 to loss and failures to diverge (Supplementary Table 3, Supplementary Table 4). These reconstructions consisted of six duplications, four host switches, three losses and finally four failures to diverge (Fig. 5).

**Figure 6:**
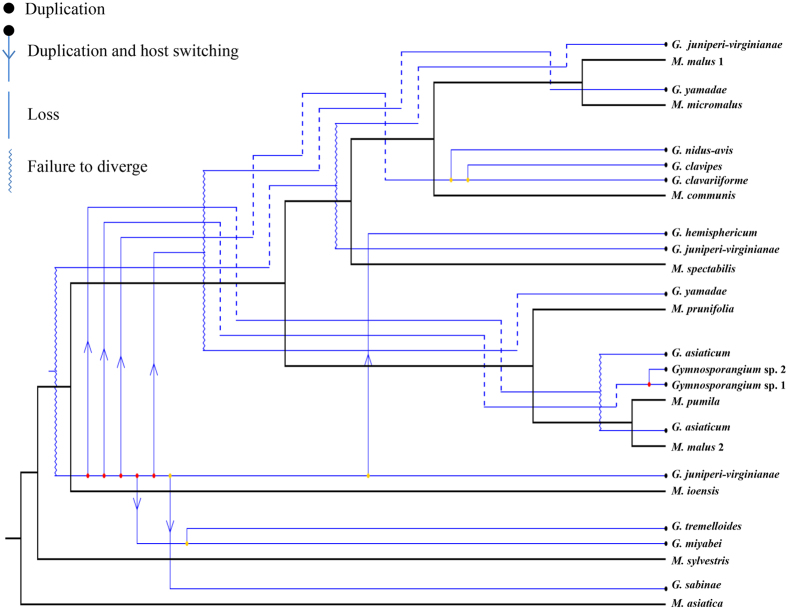
Cophylogenetic analysis of the *Gymnosporangium*–*Malus* pathosystem conducted with Jane 4 and using phylogenies including one representative per potential species of parasite and host. Black branches represent the host phylogeny and blue branches the parasite phylogeny. The cost regime used for the reconstruction was by following event costs: (cospeciation = 2, duplication = 0, host switch = 0, lineage sorting = 1 and failure to diverge = 0). The best-fit reconciliation of the *Gymnosporangium* and *Malus* trees included four duplications, seven host switches, fifteen losses and four failures to divergence.

Although we found certain codivergence between phylogenies of *Gymnosporangium* species and that of *Malus* species in Fig. 4, our results also recognized high discordance between topologies of the parasites and aecial hosts. Based on results from Jane 4.0, we recognized lowest overall costs recovered by cost regimes “6”, which assigned cost = 0 to duplication, host switch and failures to diverge (Supplementary Table 3, Supplementary Table 5). These reconstructions included four duplications, seven host switches, fifteen losses and four failures to divergence (Fig. 6).

## Discussion

Recent phylogenetic studies confirmed the monophyly of the genus *Gymnosporangium*^24^,^26^,^30^. While at the species level, there are still considerable disagreements on the delimitation of species. Our results from PTP and GMYC models recognized 14 *Gymnosporangium* species associated with its aecial host on genus *Malus*. This delimitation was supported by morphological characters from both aecial and telial stages that should be observed using dissecting microscope, light microscope and scanning electron microscope. Our results uncovered significantly higher species diversity in comparison with previous studies, which recognized one to five species from *Malus* species based on morphology from single spore stage^19^,^20^,^21^. The importance of polyphasic approaches and morphological characters from both aecial and telial spores were herein demonstrated. This result is in agreement to that of Ono *et al*.^31^, who also recognized the importance of morphological characters in both telial and aecial stages for species delimitation in rust fungi *Phakopsora ampelopsidis* complex.

The understanding of host alternation of rust fungi is important to clarify their species evolution, disease epidemics, plant quarantine and disease control. Life cycle of rust species has been previously mostly investigated based on inoculation experiments and large scale natural surveys^5^,^8^. In traditional approaches, basidiospores induced from teliospores were used as inoculums and each potential aecial and telial hosts need to be inoculated to confirm the life cycle of certain rust fungus^32^,^33^. The process of inoculation experiment is very time-consuming and laborious, and often gave false negative results because many factors, i.e., maturity of the inoculum, conditions of overwintering and inoculum germination, and growing conditions of the plants, may affect the inoculation results^33^. Although many researchers conducted studies on host alternation^5^,^11^,^34^, the life cycles from the majority of rust fungi are still unknown^35^. Taking rust fungi in Japan as an example, approximately 763 species have been recorded but the life cycles of 46% species remained unknown although systematic studies have been conducted for over a century^11^. Recently, molecular data have been used to uncover the host alternation of rust fungi, such as *Puccinia* species^36^, and *Phakopsora* species on Grape^31^. In this study, we confirmed the host alternation between *Malus* species and *Juniperus* species of 10 *Gymnosporangium* species. Among them, the host alternations of *G. juniperi-virginianae*, *G. tremelloides* and *G. yamadae*, were partially consistent with previous inoculation tests recorded by Arthur^9^, Kern^5^, Harada^33^, and Hiratsuka *et al*.^11^. Thus, the molecular phylogenetic approach, which connected the sequences obtained from telial and aecial stages respectively, is shown to be a more efficient method to determine the host alternation of rust fungi.

The evolutionary pathways of rust fungi have been categorized into four types: 1) divergence and radiation with hosts; 2) jumps to new unrelated hosts; 3) life cycle expansion; and 4) life cycle reduction^23^. The evolutionary history of *Gymnosporangium*, however, appeared to be more complicated, and different types of evolutionary patterns occurred at different time periods. Based on morphology and host range in aecial and telial stages, Leppik^37^ proposed a hypothesis that the evolution of *Gymnopsorangium* followed the life cycle reduction pattern, and the ancestral *Juniperus* species were actually aecial hosts of some heteroecious rusts. Our estimation of the divergence schedule of hosts and parasite further supported the Leppik’s hypothesis. We estimated the first common ancestor of *Gymnosporangium* species emerged approximately 51.7–44.3 Mya, but the divergent date of telia host (*Juniperus* species) appeared much earlier. The recent reports on plant fossils indicated that the divergence time of the genus *Juniperus* was 71.9–49.7 Mya during the Paleocene or adjacent periods^38^. On the contrary, the divergence of *Malus* species (approximately 31.0 Mya in Oligocene) was much later than the emergence of *Gymnosporangium* ancestor^39^.

According to Leppik^37^, the rusts on ancestral *Juniperus* species became autoecious or microcyclic by losing the connection with their telial hosts (possibly some forest ferns) when the alternate hosts were scarce or absent due to environmental change, and the rusts switched to produce urediniospores and teliospores on *Juniperus* species following “Tranzschel’s Law”^23^. Such process of evolution has already been supported by the reverse host sequence in comparison with other rust fungi, which commonly have aecial hosts on gymnosperm and telial hosts on angiosperm^7^,^22^. After adapting to new environment, *Gymnosporangium* restored the heteroecism and expanded its aecial host range to many hosts in genera of Rosaceae based on the biogenic radiation rule^40^ (Supplementary Fig. 2). This evolutionary pathway was supported by our studies because the basal linages in this genus were composed of species (*G. asiaticum* and *G. confusum*) with wide aecial hosts range up to seven genera. Our results revealed that life cycle expansion played a major role during the early evolution of *Gymnosporangium* species.

The diversification of *Gymnosporangium* species appeared to be gradually replying on their aecial hosts. According to the estimation of the divergence times of hosts and parasites, we found that early divergent *Gymnosporangium* species have a wider aecial host range at the genus level in comparison with recently divergent species. Except the uncertainty of host ranges in *Gymnosporangim* sp. 1 and *Gymnosporangim* sp. 2, our results indicated that the early divergent species group possessed aecial hosts on two to seven genera but the recent divergent species group had their aecial hosts solely on one genus, i.e. *Malus*^5^. Host ranges of *Gymnosporangium* species were shown to gradually decrease in the evolutionary process. All these species have their telial hosts on only one or two genera, and no clear tendency could be recognized. We therefore speculated that the aecial hosts played more important roles than telial hosts in the evolution and speciation of *Gymnosporangium* species.

According to the analysis of cophylogeny at species level, the driving forces of speciation appeared to be more complicated. Previously, host switch was considered to be difficult for *Gymnopsorangium* species because their life cycles are mainly restricted to Cupressaceae and Rosaceae^8^. Most *Gymnopsorangium* species were thought to be tightly coevolved with their host species albeit lack of the overall cospeciation model. Novick^26^ however, raised a different opinion that host switch was a main speciation pattern. According to Johnson *et al*.^41^, the processes in host–parasite coevolutionary histories could be divided into eight different events: cospeciation, failure to speciate, duplication, extinction, missing the boat, incomplete host switching, host switch with extinction, host switch with speciation, host switch with speciation and extinction. Through the molecular clock analyses, McTaggart *et al*.^24^ concluded that host jumps, rather than coevolution, were the main speciation events that drove the diversification of rust fungi at family and genus level. They also suspected that cospeciation and host switch might be main force at species level. However, we did not recognize cospeciation event of *Gymnosporangium* species with its aecial and telial hosts, although codivergences were recognized in the tanglegram of host and parasite phylogenies. Our results revealed that host switch, duplication, losses and failure to divergence all played certain roles in driving the speciation in both *Juniperus*-parasite system and *Malus*-parasite system. Among them, the process of host switch with speciation and extinction appeared to play the most important roles in the evolutionary history of *Gymnosporangium*-*Malus* system. The noteworthy multiple speciation mechanisms existed in this group might be a reflection of their complicated life cycles, as compared to other phytophathogenic fungi. It is therefore very interesting for future studies to investigate why diversified speciation mechanisms exist in the genus *Gymnopsorangium*.

## Materials and Methods

### Fungal specimens

A total of 173 herbarium specimens were loan from the Mycological Herbarium of Institute of Microbiology, CAS, China (**HMAS**) to cover the largest possible hosts and localities of *Gymnosporangium* species based on previous taxonomic literatures^19^,^20^,^21^. Specimens with either aecial or telial stage were chosen according to the names on the attached labels and their host information. Among these, 72 specimens were collected from *J. chinensis* and 101 specimens were collected from *Malus* species. In addition, 85 specimens, which were labeled as *Gymnosporangium* species on *Malus* species and *Juniperus* species from Europe and North America, were loaned from following herbaria: Plant Pathology Herbarium, Cornell University, Ithaca, New York, USA (**CUP**); and New York Botanical Garden, New York, USA (**NYBG**). The detailed information of specimens used in this study is listed in Supplementary Table 6.

### DNA extraction, sequencing and phylogenetic analyses

For the fungal specimens, single sorus from each specimen was excised and DNAs were extracted from all studied herbarium specimens by using Gentra Puregene Tissue Kit (Qiagen, Valencia, CA) according to the manufacturer’s instructions. From the crude extracts, 1 to 3 μl DNA templates were used directly for the polymerase chain reaction (PCR) amplification of the internal transcribed spacer regions and intervening 5.8S nrRNA gene (ITS) and the large subunit (LSU) rDNA, and nested PCR method was employed to improve the amplification. The detailed information of primers was listed in the Supplementary Table 7, and the annealing temperatures of these target fragments were followed by Beenken *et al*.^42^.

The rDNA ITS and LSU were successfully obtained from 72 herbarium specimens, and their herbarium number, host species, geographical origins and GenBank accession numbers were shown in Supplementary Table 6. Additional 42 sequence data of *Gymnospornagium* species, which including both rDNA ITS regions and LSU, were retrieved from GenBank for comparable studies (Supplementary Table 8). Two sequences of *Ravenelia* species were selected as the outgroups. A dataset was constructed including 114 sequence data. Sequences were manually aligned by using Bioedit v.7.0.9^43^, and multiple alignments were performed in Clustal X v.1.8^44^. Gaps were treated as missing data for all analyses. The Akaike Information Criteria (AIC) in Modeltest v.3.7^45^ was used to estimate the best-fit substitution models. Maximum Likelihood (ML) analyses were performed using RAxML v.8.0.X^46^, and Bayesian Markov chain Monte Carlo (MCMC) analyses were performed by MrBayes v.3.1.2^47^.

### GMYC and PTP analyses

Species were recognized based on the concordant results from GMYC and PTP models. The GMYC model uses the distinct branching patterns between a Yule model (modeling interspecific speciation events) and a coalescent model (modeling expected coalescent times of genes at an intraspecific level) to distinguish between species^48^. Thus, GMYC model was employed to delimit phylogenetic species. Ultrametric trees required to rim the GMYC algorithm were created using BEAST ver. 1.7.5^49^. Two sets of analyses, single-threshold and multiple-threshold models, were performed in the GMYC, and three independent MCMC analyses were run for 100 million generations, sampling trees every 10,000 generations. The posterior tree was summarized using TreeAnnotator after discarding burn-in which was determined by Tracer ver. 1.6^50^. The selected topologies were used to optimize the single- and multiple-threshold GMYC models, using the ‘splits’ package^51^ available for R 3.0.2 (R Core Team 2013). At last, the program STEM was used to estimate likelihood scores of alternative species delimitation scenarios obtained from single- and multiple-threshold GMYC^52^, and the putative species scenario was selected based on the value of estimated likelihood scores according to Carstens & Dewey^53^.

As to the species delimitation by PTP model, it uses the branch lengths to estimate the mean expected number of substitutions per site between two branching events^54^. The model assumes that each substitution has a small probability of generating a speciation event, the model then implements two independent classes of Poisson processes (one describing speciation and the other describing within species branching events) and searches for transition points between interspecific and intraspecific branching patterns. Potential species clusters are then determined by identifying the clades (or single lineages) that originate after these transition points^54^. The analyses were conducted on the web server for PTP (available at http://species.h-its.org/ptp/) using the RAxML topology as advocated for this method^54^.

In this study, the species boundary was determined based on consistent results obtained from GMYC and PTP models. Thereafter, the detailed morphological characters of each specimen were observed under the dissecting microscope (SMZ745, Nikko, Japan), the light microscope (Axio Imager A2, ZEISS, Germany) and the scanning electron microscope (Quanta 200, FEI^TM^, USA). The proper name of each putative species was determined based mainly on the keys developed by Kern^5^ and Peterson^8^. Besides, morphological characteristics were compared with the original descriptions, and other published descriptions of species involved^5^,^9^,^10^,^12^,^27^,^29^.

### Molecular clock analysis

To determine the evolutionary history of *Gymnosporangium* species, the *Ravenelia* (Raveneliaceae, Pucciniales) fossil was selected as a reliable calibration point to estimate the divergence time of *Gymnosporangium* species in the Pucciniales, and this genus was separated from other members of the rust fungi at the minimum ages of 55.8–40.4 Mya^55^. The calculations of molecular clock dated linearized BI trees were performed in BEAST v.1.7.5 with the concatenated sequences of rDNA ITS regions and LSU. BEAST input files were constructed using BEAUti (within BEAST), and the lognormal relaxed molecular clock model and the Yule speciation prior set were used to estimate the divergence times and the corresponding credibility intervals. FigTree v.1.4.0 was used to visualize the resulting tree and to obtain the means and 95% higher posterior densities (HPD).

### Cospeciation analysis

To conduct the cospeciation analyses, we tried to extract DNA from aecial and telial host species. Gentra Puregene Tissue Kit was used to extract DNA from plant tissues and rDNA ITS and the large subunit of the ribulose-bisphosphate carboxylase gene (rbcL) were selected for amplification. Detailed information of primers and PCR procedures was shown in Supplementary Table 7, and information of specimens and their GenBank accession No. was also shown in Supplementary Table 9. PCR amplification procedure and primers of Robinson *et al*.^1^ were used to amplify these two target regions from *Malus* species. In addition, the same primers and PCR procedure of Adams *et al*.^56^, were employed to amplify target regions from *Juniperus* species. Due to limited sequence variations of rbcL gene among host species, especially *Malus* species, we only used rDNA ITS regions from host species for further cophylogenetic analyses.

Cospeciation analysis was conducted based on an event-cost based test of cospeciation, and Treemap 3.0ß and Jane 4.0^57^ were employed to test for significant congruence between the *Gymnosporangium* and its aecial and telia host’s topologies, respectively. The tanglegram from the phylogenetic trees and individual associations were built by Treemap. In the event cost based tests implemented by Jane 4.0, a pruned ITS regions and nLSU alignment including only one representative per putative species of hosts and parasites was used for analyses. Cophylogeny mapping in Jane 4.0 used heuristics to reconstruct histories that explain the similarities and differences between associated phylogenies. It prioritized minimizing the overall cost, given a cost regime for evolutionary events including “cospeciation”, “duplication”, “duplication and host switch”, “loss” or “lineage sorting”, and “failure to diverge”^57^,^58^. In all analyses the number of generations was set to 100, and the population size to 300, with a maximum of 99,999 stored solutions in each run. Statistical analyses were then performed to test whether the cost of the reconstructions obtained was significantly lower than expected by chance. Jane 4.0 was used to generate a pseudorandom sample of minimal costs from a null distribution of problem instances with the same model phylogeny. The null distribution was generated by repeatedly randomizing the host–parasite associations (Random Tip Mapping), and the significant matching of host and parasite phylogenies was evaluated by computing the costs of 1000 replicates to compare the resulting costs to the cost of the original cophylogeny.

## Additional Information

**How to cite this article**: Zhao, P. *et al*. Inferring phylogeny and speciation of *Gymnosporangium* species, and their coevolution with host plants. *Sci. Rep.* **6**, 29339; doi: 10.1038/srep29339 (2016).

## Supplementary Material

Supplementary Information

## References

[b1] Robinson J. P., Harris S. A. & Juniper B. E. Taxonomy of the genus *Malus* Mill. (Rosaceae) with emphasis on the cultivated apple, *Malus domestica* Borkh. Plant Syst. Evol. 226, 35–58 (2001).

[b2] Li B. H., Wang C. X. & Dong X. L. Research progress in apple diseases and problems in the disease management in China (in Chinese). Plant Prot. 39, 46–54 (2013).

[b3] Sinclair W. A. & Lyon H. H. Diseases of trees and shrubs. 2^nd^ ed. Ithaca, New York, USA: Cornell University Press. (2005).

[b4] Lamarck J. B. & de Candolle A. P. Flore française. 2, 1–600 (1805).

[b5] Kern F. D. A revised taxonomic account of Gymnosporangium. University Park, Pennsylvania, USA: Pennsylvania State University Press. (1973).

[b6] Farr D. F. & Rossman A. Y. Fungal Databases, Systematic Mycology and Microbiology Laboratory, ARS, USDA. Available at: http://nt.ars-grin.gov/fungaldatabases/. (Accessed: 20 October 2015) (2013).

[b7] Cummins G. B. & Hiratsuka Y. *Illustrated genera of rust fungi, 3*^*rd*^ *edn*. St. Paul, Minnesota, USA: American Phytopathological Society. (2003).

[b8] Peterson R. S. Rust fungi (Uredinales) on Cupressaceae. Mycologia 74, 903–910 (1982).

[b9] Arthur J. C. Manual of the rusts in United States and Canada. West Lafayette, Indiana, USA: Purdue Research Foundation. (1962).

[b10] Wilson M. & Henderson D. M. The British rust fungi. Cambridge, UK: Cambridge University Press. (1966).

[b11] Hiratsuka N. . *The rust flora of Japan*. Tsukuba, Ibaraki, Japan: Tsukuba: Shuppankai,. (1992).

[b12] Sydow P. & Sydow H. Monographia Uredinearum. III: Melampsoraceae, Zaghouaniaceae, Coleosporiaceae. Berlin, Germany: Borntraeger, Leipzig. (1915).

[b13] Virtudazo E. V., Nakamura H. & Kakishima M. Phylogenetic analysis of sugarcane rusts based on sequences of ITS, 5.8 S rDNA and D1/D2 regions of LSU rDNA. J. Gen. Plant Pathol. 67, 28–36 (2001).

[b14] Tian C. M., Shang Y. Z., Zhuang J. Y., Wang Q. & Kakishima M. Morphological and molecular phylogenetic analysis of *Melampsora* species on poplars in China. Mycoscience 45, 55–66 (2004).

[b15] Pons J. . Sequence-based species delimitation for the DNA taxonomy of undescribed insects. Syst. Biol. 55, 595–609 (2006). 16967577 10.1080/10635150600852011

[b16] Parnmen S. . Using phylogenetic and coalescent methods to understand the species diversity in the *Cladia aggregata* complex (Ascomycota, Lecanorales). PLoS ONE 7, e52245 (2012). 23272229 10.1371/journal.pone.0052245 PMC3525555

[b17] Singh G. . Coalescent-based species delimitation approach uncovers high cryptic diversity in the cosmopolitan lichen-forming fungal genus *Protoparmelia* (Lecanorales, Ascomycota). PLoS ONE 10, e0124625 (2015). 25932996 10.1371/journal.pone.0124625 PMC4416777

[b18] Sawada K. Descriptive catalogue of the Formosan fungi IV. Report of the Department of Agriculture Government Research Institute of Formosa 35, 1–123 (1928).

[b19] Tai F. L. Sylloge Fungorum Sinicorum (in Chinese). Beijing, China: Science Press. (1979).

[b20] Zhuang W. Y. Fungi of northwestern China. : Ithaca, New York, USA, : Mycotaxon Ltd. (2005).

[b21] Zhuang J. Y., Wei S. X. & Wang Y. C. Flora fungorum sinicorum. Vol. 41. Uredinales IV. Beijing, China: Science Press. (2012).

[b22] Leppik E. E. Some viewpoints on the phylogeny of rust fungi. II. Gymnosporangium. Mycologia 48, 637–654 (1956).

[b23] Hennen J. F. & Buriticá P. A. A brief summary of modern rust taxonomic and evolutionary theory. Rep. Tottori Mycol. Inst. 18, 243–256 (1980).

[b24] McTaggart A. R. . Host jumps shaped the diversity of extant rust fungi (Pucciniales). New Phytol. 10.1111/nph.13686 (2016). 26459939

[b25] Moran N. Benefits of host plant specificity in *Uroleucon* (Homoptera: Aphididae). Ecology 67, 108–115 (1986).

[b26] Novick, R. S. Phylogeny, taxonomy, and life cycle evolution in the cedar rust fungi (*Gymnosporangium*). PhD Thesis, Yale University. New Haven. Connecticut, USA (2008).

[b27] Kuprevich V. F. & Tranzschel V. G. Rust fungi. 1. Family Melampsoraceae. (ed. Savich V. P.) Cryptogamic plants of the USSR. vol 4. Komarova, Russia: Botanicheskogo Instituta, 423–464 (1957).

[b28] Lee S. K. & Kakishima M. Surface structures of peridial cells of *Gymnosporangium* and *Roestelia* (Uredinales). Mycoscience 40, 121–131 (1999).

[b29] Yun H. Y. . The rust fungus *Gymnosporangium* in Korea including two species *G. monticola* and *G. unicorne*. Mycologia 1, 790–809 (2009). 19927745 10.3852/08-221

[b30] Maier W., Begerow D., Weiss M. & Oberwinkler F. Phylogeny of the rust fungi: an approach using nuclear large subunit ribosomal DNA sequences. Can. J. Bot. 81, 12–23 (2003).

[b31] Ono Y., Chatasiri S., Pota S. & Yamaoka Y. *Phakopsora montana*, another grapevine leaf rust pathogen in Japan. J. Gen. Plant Pathol. 78, 338–347 (2012).

[b32] Parmelee J. A. Gymnosporangium juniperi-virginianae. Fungi Canadensis 137, 1–2 (1979).

[b33] Harada Y. Pear and apple rusts in Japan, with special reference to their life cycles and host ranges. Rep. Tottori Mycol. Inst. 22, 108–119 (1984).

[b34] Klebahn H. *Uredineae* (in German). *Kryptogamenflora der Mark Brandenburg Va*. Berlin, Germany: Leipzig Gebrüder Borntraeger. (1914).

[b35] Arthur J. C. The Uredinales (rusts) of Iowa. Bulletin of Iowa Academy of Science 31, 229–255 (1924).

[b36] Anikster Y. . Morphology, life cycle biology, and DNA sequence analysis of rust fungi on garlic and chives from California. Phytopathology 94, 569–577 (2004). 18943481 10.1094/PHYTO.2004.94.6.569

[b37] Leppik E. E. Origin and evolution of conifer rusts in the light of continental drift. Mycopathologia et Mycologia Applicata 49, 121–136 (1973).

[b38] Mao K. S., Hao G., Liu J. Q., Adam R. P. & Milne R. I. Diversification and biogeography of *Juniperus* (Cupressaceae): variable diversification rates and multiple intercontinental dispersals. New Phytol. 188, 254–272 (2012). 20561210 10.1111/j.1469-8137.2010.03351.x

[b39] Meyer H. W. & Manchester S. R. The Oligocene bridge creek flora of the John Day formation, Oregon.v14. California, USA: University of California Press. (1997).

[b40] Leppik E. E. Some viewpoints on the phylogeny of rust fungi. IV. Biogenic radiation. Mycologia 59, 568–579 (1967).

[b41] Johnson K. P., Adams R. J., Page R. D. M. & Clayton D. H. When do parasites fail to speciate in response to host speciation? Syst. Biol. 52, 37–47 (2003). 12554438 10.1080/10635150390132704

[b42] Beenken L., Zoller S. & Berndt R. Rust fungi on Annonaceae II: the genus *Dasyspora* Berk. & M. A. Curtis. Mycologia 104, 659–681 (2012). 22223173 10.3852/11-068

[b43] Hall T. A. BioEdit: a user-friendly biological sequence alignment editor and analysis program for Windows 95/98/NT. Nucleic Acids Symp. Ser. 41, 95–98 (1999).

[b44] Thompson J. D., Gibson T. J., Plewniak F., Jeanmougin F. & Higgins D. G. The CLUSTAL_X windows interface: flexible strategies for multiple sequence alignment aided by quality analysis tools. Nucleic Acids Res. 25, 4876–4882 (1997). 9396791 10.1093/nar/25.24.4876 PMC147148

[b45] Posada D. & Crandall K. A. MODELTEST: testing the model of DNA substitution. Bioinformatics 14, 817–818 (1998). 9918953 10.1093/bioinformatics/14.9.817

[b46] Stamatakis A. RAxML version 8: a tool for phylogenetic analysis and post-analysis of large phylogenies. Bioinformatics. 10.1093/bioinformatics/btu033 (2014). PMC3998144 24451623

[b47] Huelsenbeck J. P. & Ronquist F. Mrbayes: Bayesian inference of phylogenetic trees. Bioinformatics 17, 754–755 (2001). 11524383 10.1093/bioinformatics/17.8.754

[b48] Fujisawa T. & Barraclough T. G. Delimiting species using single-locus data and the generalized mixed yule coalescent (GMYC) approach: a revised method and evaluation on simulated datasets. Syst. Biol. 1, 1–18 (2013). 10.1093/sysbio/syt033 PMC3739884 23681854

[b49] Drummond A. J. & Rambaut A. BEAST: Bayesian evolutionary analysis by sampling trees. BMC Evol. Biol. 7, 214 (2007). 17996036 10.1186/1471-2148-7-214 PMC2247476

[b50] Rambaut A., Suchard M. A., Xie D. & Drummond A. J. Tracer v1. 6. Available at: http://beast.bio.ed.ac.uk/Tracer. (Accessed: 1st August 2015) (2014).

[b51] Ezard T., Fujisawa T. & Barraclough T. Species limits by threshold statistics. Available at: http://R-Forge.R-project.org/projects/splits/. (Accessed: 21st August 2015) (2009).

[b52] Kubatko L. S., Carstens B. C. & Knowles L. L. STEM: species tree estimation using maximum likelihood for gene trees under coalescence. Bioinformatics 1, 1–3 (2009). 10.1093/bioinformatics/btp079 19211573

[b53] Carstens B. C. & Dewey T. A. Species delimitation using a combined coalescent and information-theoretic approach: an example from North American myotis bats. Syst. Biol. 59, 400–414 (2010). 20547777 10.1093/sysbio/syq024 PMC2885268

[b54] Zhang J. J., Kapli P., Pavlidis P. & Stamatakis A. A. General species delimitation method with applications to phylogenetic placements. Bioinformatics 29, 2869–2876 (2013). 23990417 10.1093/bioinformatics/btt499 PMC3810850

[b55] Ramanujan C. K. & Ramachar P. Spore dispersal of the rust fungi (Uredinales) from the Miocene lignite of South India. Curr. Sci. 32, 271–272 (1963).

[b56] Adams R. P. & Schwarzbach A. E. Phylogeny of *Juniperus* using nrDNA and four cpDNA regions. Phytologia 95, 179–185 (2013).

[b57] Conow C., Fielder D., Ovadia Y. & Libeskind-Hadas R. Jane: a new tool for the cophylogeny reconstruction problem. Algorithms Mol. Biol. 5, 16 (2010). 20181081 10.1186/1748-7188-5-16 PMC2830923

[b58] Charleston M. A. Jungles: a new solution to the host/parasite phylogeny reconciliation problem. Math. Biosci. 149, 191–223 (1998). 9621683 10.1016/s0025-5564(97)10012-8

