# Supplementary material for: Inferring phylogeny and speciation of Gymnosporangium species, and their coevolution with host plants
Source: Sci Rep. 2016 Jul 7;6:29339. doi: 10.1038/srep29339 (PMC4935989; doi:10.1038/srep29339)
Supplement: Supplementary Information [file srep29339-s1.pdf]

SUPPLEMENTARY INFORMATION

Inferring phylogeny and speciation of *Gymnosporangium* species, and their coevolution with host plants

Peng Zhao, Fang Liu, Ying-Ming Li & Lei Cai*

State Key Laboratory of Mycology, Institute of Microbiology, Chinese Academy of Sciences, Beijing 100101, China

Supplementary info:

1. Supplementary Table 1. Morphological characteristics of *Gymnosporangium* species recognized in this study
2. Supplementary Table 2. Host alternation of putative *Gymnosporangium* species recognized in this study
3. Supplementary Table 3. Cost regimes used in the cophylogeny analyses using Jane 4.0
4. Supplementary Table 4. Numbers of the different evolutionary events inferred in order to reconcile *Gymnosporangium* and *Juniperus* phylogenies
5. Supplementary Table 5. Numbers of the different evolutionary events inferred in order to reconcile *Gymnosporangium* and *Malus* phylogenies
6. Supplementary Table 6. Sequenced specimens of *Gymnosporangium* and their GenBank accession numbers of rDNA ITS regions and 28S obtained from this study
7. Supplementary Table 7. Primers sequences and PCR conditions used to amplify target regions from *Gymnosporangium* and its host species
8. Supplementary Table 8. GenBank accession numbers of *Gymnosporangium* species the downloaded sequences used in the phylogenetic analyses
9. Supplementary Table 9. GenBank accession numbers of rDNA ITS regions of *Malus* species and *Juniperus* species obtained from this study
10. Supplemental Figure 1 - Morphological variations of *Gymnopsorangium* species in aecial and telia stage. (A) roestelioid aecia. (B) aecidioid aecia. (C) peridium with cornuted apex. (D) peridium with tubular apex. (E) oblonged or oval peridium cell. (F) rhomboidal perdium cell. (G) peridium cell verruculose with ridge-like papillae. (H) peridium cell verruculose with large papillae. (I) peridium cell verruculose. (J) globoid or ellipsoid aeciospores with 3 to 5 germ pores. (K) aeciospores with large coronate spine on the surface. (L) aeciospores with large echinulate spine on the surface. (M) fusiform and wedge-shaped telia. (N) witches' broom and hemispheric telia. (O) terete and fusiform telia. (P) witches' broom and conical telia. (Q) slight fusiform and applanate telia. (R) gall-type telia with irregular sori. (S) gall-type telia with wedge-shaped sori. (T) gall-type telia with cylindric-acuminate sori. (U) 2-celled teliospores with long and cylindrical perdicels. (V) 1 to 4 celled teliospores with long and cylindrical perdicels. (W) 2-celled teliospores narrowed above and below and with long and cylindrical perdicels. (X) 2-celled teliospores with carotiform type perdicles. Bars:

A, B, C, D = 2 mm; E, F = 40 μm; G, I, K, L =10 μm; H = 2 μm; J = 20 μm; M, N, O, P, R, S = 1cm; U, V, W, X = 50 μm.

11. Supplementary Figure 2 - Hypothesis of the evolutionary process of *Gymnosporangium* species on *Malus*. The alphabets S, A, U and T represent spermogonia, aecia, uredinia and telia in the whole life cycle, respectively. The asterisk (*) represents the reduced life cycle of the ancestor of *Gymnopsorangium* species based on the Tranzschel's Law. The question mark (?) indicates that some forest ferns were speculated as the host species of the ancestor of *Gymnosporangium* species, and uredinial and telial stages might occurred on ferns. The epochs in the parentheses indicate the estimated time period when the evolutionary pathway of rust fungi occurred.

Supplementary Table 1. Morphological characteristics of *Gymnosporangium* species recognized in this study

Species	Morphology in aecial stage								Morphology in telial stage			
	Shape of aecia	Length of peridium (mm)	Apex of peridium	Shape of peridium cells	Ornaments of peridium cells	Dimension of peridium cells (µm)	Oramentation of aeciospores	Dimension of aeciospores (µm)	Shape of telia	Cell of teliospores	Dimension of teliospores (µm)	Shape of perdicels
G. asiaticum	roestelioid	4.0–7.0	tubular	rhomboidal	verruculose	55–103 × 18–31	large coronate spine	18–26 × 14–22	terete, fusiform	2	31–50 × 16–27	long, cylindrical
G. clavariiforme	roestelioid	1.5–2.0	tubular	rhomboidal	verruculose	77–148 × 15–29	echinulate spine	16–30 × 18–25	terete, fusiform	2	46–97 × 15–25	long, cylindrical
G. clavipes	roestelioid	1.5–3.5	tubular	rhomboidal	verruculose with large papillae	48–71 × 21–42	echinulate spine	26–45 × 23–33	witches' broom, hemispheric	2	25–58 × 13–30	carotiform
G. globosum	ND*	ND	ND	ND	ND	ND	ND	ND	gall, sori irregular	2	40–54 × 15–23	long, cylindrical
G. hemisphericum	roestelioid	1.5–2.5	tubular	rhomboidal	verruculose with ridge-like papillae	63–88 × 24–37	large coronate spine	23–35 × 17–31	witches' broom, conical	2	22–41 × 15–28	long, cylindrical
G. juniperi-virginianae	aecidioid	0.2–0.5	tubular	rhomboidal	verruculose with large papillae	56–109 × 19–34	coronate spine	18–33 × 13–26	gall, sori cylindric-acuminate	2	35–72 × 17–26	long, cylindrical
G. miyabei	roestelioid	2.0–3.5	cornute	oblong	verruculose	62–113 × 37–52	coronate spine	22–39 × 15–28	slight fusiform, applanate	1 to 3	38–93 × 12–25	long, cylindrical
G. nelsonii	ND	ND	ND	ND	ND	ND	ND	ND	gall, sori wedge-shaped	2	45–77 × 23–32	long, cylindrical
G. nidus-avis	roestelioid	2.0–4.5	tubular	rhomboidal	verruculose with ridge-like papillae	40–121 × 23–58	coronate spine	18–35 × 19–25	witches' broom, hemispheric	1 to 4	32–55 × 18–24	long, cylindrical
G. sabinae	roestelioid	3.5–6.0	cornute	rhomboidal	verruculose	51–87 × 19–32	coronate spine	21–32 × 17–31	terete, fusiform	2	38–44 × 16–23	long, cylindrical
G. tremelloides	roestelioid	1.5–2.5	tubular	rhomboidal	verruculose with ridge-like papillae	69–105 × 33–51	echinulate spine	27–49 × 24–37	slight fusiform, applanate	2	32–85 × 23–32	long, cylindrical
G. yamadae	roestelioid	4.5–8.5	tubular	rhomboidal	verruculose	43–99 × 18–30	coronate spine	17–28 × 15–24	gall, sori cylindric-acuminate	2	26–52 × 13–25	long, cylindrical
***Gymnosporangium* sp.1**	roestelioid	1.5–3.5	cornute	oblong	verruculose	58–72 × 24–37	coronate spine	15–26 × 15–23	ND	ND	ND	ND
***Gymnosporangium* sp.2**	roestelioid	2.0–4.5	cornute	rhomboidal	verruculose with ridge-like papillae	36–58 × 16–22	coronate spine	23–35 × 21–27	ND	ND	ND	ND

Supplementary Table 2. Host alternation of putative *Gymnosporangium* species recognized in this study

***Gymnosporangium* species**	**Aecial host**	**Telial host**
G. juniperi-virginianae	*Malus malus, M. ioensis*	*Juniperus virginiana*
G. hemisphaericum	*M. spectubilis*	*J. chinensis, J. virginiana*
G. yamadae	*M. prunifolia, M. micromalus*	*J. chinensis*
G. globosum	ND[a]	*J. chinensis, J. scopulorum, J. virginiana*
G. nelsonii	ND	*J. horizontalis*
G. sabinae	*M. asiatica*	*J. chinensis, J. virginiana*
G. miyabei	*M. sylverstris*	*J. chinensis, J. virginiana*
G. tremelloides	*M. sylvestris*	*J. communis* var. *depressa*
Gymnosporangium sp.1	*M. pumila*	ND
Gymnosporangium sp.2	*M. pumila*	ND
G. nidus-avis	*M. communis*	*J. chinensis, J. horizontalis, J. virginiana*
G. clavipes	*M. communis*	*J. communis*
G. clavariiforme	*M. communis*	*J. communis*
G. asiaticum	*M. pumila, M. malus*	*J. chinensis*

a: ND indicated that the host species was not detected in this study.

Supplementary Table 3. Cost regimes used in the cophylogeny analyses using Jane 4.0

	Cost assigned to each event				
	Cospeciation	Duplication	Host switch	Loss	Failure to diverge
Cost regime 1	0	1	2	1	1
Cost regime 2	1	1	1	1	1
Cost regime 3	1	0	0	1	1
Cost regime 4	1	0	0	1	0
Cost regime 5	2	0	0	1	1
Cost regime 6	2	0	0	1	0
Cost regime 7	2	0	1	1	1
Cost regime 8	2	0	2	1	0
Cost regime 9	2	0	2	1	1
Cost regime 10	2	0	2	2	1

Supplementary Table 4. Numbers of the different evolutionary events inferred in order to reconcile *Gymnosporangium* and *Juniperus* phylogenies

	**Cospeciation**	**Duplication**	**Host switch**	**Loss**	**Failure to diverge**	**Number of events**	**P[a]**
Cost regime 1	3	2	5	4	4	20	0.02*
Cost regime 2	0	6	4	3	4	17	0.03**
Cost regime 3	0	6	4	3	4	7	0.02*
Cost regime 4	0	6	4	3	4	28	0.1*
Cost regime 5	0	6	4	3	4	7	0.02*
Cost regime 6	0	6	4	3	4	7	0.03**
Cost regime 7	0	6	4	3	4	11	**0.008****
Cost regime 8	0	6	4	3	4	11	0.049*
Cost regime 9	0	6	4	3	4	15	0.03**
Cost regime 10	0	6	4	3	4	18	0.018**

a: *P*-values of each randomized test using Random Tip Mapping (RTM) method are indicated. Significant *P*-values are highlighted in bolds and with asterisks (∗*P*-value < 0.05 and ∗∗*P*-value < 0.01).

Supplementary Table 5. Numbers of the different evolutionary events inferred in order to reconcile *Gymnosporangium* and *Malus* phylogenies

	Cospeciation	Duplication	Host switch	Loss	Failure to diverge	Number of events	P[a]
Cost regime 1	0	9	2	16	4	33	0.05*
Cost regime 2	0	4	7	15	4	30	0.08*
Cost regime 3	0	4	7	15	4	19	0.008**
Cost regime 4	0	4	7	15	4	15	0.005**
Cost regime 5	0	4	7	15	4	19	0.04*
Cost regime 6	0	4	7	15	4	15	**0.002****
Cost regime 7	0	9	2	16	4	22	0.3
Cost regime 8	0	9	2	16	4	20	0.07*
Cost regime 9	0	9	2	16	4	22	0.1*
Cost regime 10	0	9	2	16	4	40	0.4

a: *P*-values of each randomized test using Random Tip Mapping (RTM) method are indicated. Significant *P*-values are highlighted in bolds and with asterisks (∗*P*-value < 0.05 and ∗∗*P*-value < 0.01).

Supplementary Table 6. Sequenced specimens of *Gymnosporangium* and their GenBank accession numbers of rDNA ITS regions and 28S obtained from this study

Spore stage	Host plants	Specimen no[a].	Locality[b]	GenBank accession no.	
				rDNA ITS regions	28S
Aecial stage	*M. asiatica*	HMAS17707	China, Beijing	KU288688	KU342759
		HMAS140407	China, Beijing	KU288648	KU342741
	M. communis	NYBG237060	USA, Kansas	KU288706	KU342747
		CUP43859	USA, Massachusetts	KU288680	KU342769
		CUP21441	USA, New York	KU288635	KU342761
	M. ioensis	CUP595	USA, New York	KU288676	KU342709
	M. malus	CUP20165	USA, New York	KU288677	KU342708
		CUP20159	USA, New York	KU288673	KU342711
		CUP20204	USA, New York	KU288670	KU342715
		CUP20257	USA, Tennessee	KU288678	KU342716
		HMAS135289	China, Yunnan	KU288657	KU342773
	M. micromalus	HMAS199333	China, Beijing	KU288674	KU342724
		HMAS246995	China, Beijing	KU288666	KU342725
		HMAS243188	China, Beijing	KU288659	KU342726
		HMAS246997	China, Inner Mongolia	KU288660	KU342728
		HMAS246998	China, Beijing	KU288638	KU342729
	M. prunifolia	HMAS36992	China, Gansu	KU288662	KU342732
		HMAS157685	China, Shaanxi	KU288698	KU342734
	M. pumila	HMAS55757	China, Beijing	KU288691	KU342749
		HMAS38649	China, Shaanxi	KU288690	KU342752
		HMAS246994	China, Inner Mongolia	KU288692	KU342751
		HMAS246996	China, Inner Mongolia	KU288693	KU342750
		HMAS44514	China, Sichuan	KU288696	KU342753
		HMAS44394	China, Sichuan	KU288683	KU342754
		HMAS14328	China, Jiangsu	KU288661	KU342772
		HMAS14327	China, Hebei	KU288671	KU342775
		HMAS44513	China, Hebei	KU288704	KU342778
	M. spectabilis	HMAS26416	China, Sichuan	KU288682	KU342723
		HMAS12940	China, Beijing	KU288667	KU342735
	M. sylvestris	HMAS70746	Finland, Nyland	KU288645	KU342747
		CUP56165	Finland, Aland	KU288675	KU342748
Telial stage	*J. chinensis*	HMAS79065	China, Guizhou	KU288684	KU342721
		HMAS79186	China, Beijing	KU288669	KU342722
		HMAS82779	China, Inner Mongolia	KU288646	KU342730
		HMAS8632	China, Beijing	KU288702	KU342777
		HMAS55353	China, Beijing	KU288695	KU342727
		HMAS11216	China, Jiangsu	KU288685	KU342733
		HMAS135611	China, Hubei	KU288699	KU342736
		HMAS47228	China, Guangdong	KU288642	KU342774
		HMAS47229	China, Beijing	KU288643	KU342776
		HMAS172366	China, Beijing	KU288703	KU342779
		HMAS165302	China, Yunnan	KU288658	KU342771
	J. communis	HMAS2146	Scandinavia	KU288679	KU342767
		HMAS24626	Canada, Ontario	KU288672	KU342766
		HMAS67951	Germany, Bayem	KU288644	KU342765
		NYBG23203	USA, New York	KU288639	KU342768
	J. horizontalis	NYBG193243	USA, California	KU288653	KU342739

	NYBG193254	USA, California	KU288663	KU342740
	NYBG33324	USA, Iowa	KU288636	KU342760
J. sabina	HMAS143609	China, Guizhou	KU288649	KU342742
J. virginiana	HMAS8974	China, Anhui	KU288640	KU342707
	HMAS14419	China,Jiangsu	KU288681	KU342756
	HMAS14315	USA, Iowa	KU288641	KU342745
	NYBG23036	USA, California	KU288637	KU342710
	NYBG1391099	USA, North California	KU288647	KU342712
	NYBG237085	USA, Iowa	KU288654	KU342713
	NYBG18554	USA, Kansas	KU288664	KU342714
	NYBG665	USA, California	KU288689	KU342717
	NYBG237070	USA, Kansas	KU288656	KU342718
	NYBG237046	China, Iowa	KU288655	KU342719
	NYBG237061	USA, Kansas	KU288650	KU342720
	NYBG237038	USA, Kansas	KU288651	KU342738
	NYBG461220	USA, Massachusetts	KU288652	KU342743
	NYBG237094	USA, New Mexico	KU288686	KU342755
	NYBG237060	USA, Kansas	KU288694	KU342759
	NYBG237080	Canada, Saskatchewan	KU288700	KU342757
	NYBG461234	Canada, Ontario	KU288701	KU342758
	NYBG237077	USA, Kansas	KU288697	KU342746
	NYBG237071	USA, Kansas	KU288688	KU342762
	NYBG237072	USA, Kansas	KU288687	KU342763

[a]**HMAS**: the Mycological Herbarium of Institute of Microbiology, CAS, China; **CUP**: Plant Pathology Herbarium, Cornell University, Ithaca, NY, USA; **NYBG**: New York Botanical Garden, NY, USA.

[b]Locality with name of country followed by the name of provinces or prefectures.

[c]ND: Not analyzed.

Supplementary Table 7. Primers sequences and PCR conditions used to amplify target regions from *Gymnosporangium* and its host species

**Materials**	**Loci**	**Primers (references*)**	**PCR thermo cycling locus-specific conditions**
Gymnosporangium species	ITS1-5.8S-ITS2 (600 bp)	Rust2inv (Aime 2006)/Gym-reverse (Yun et al. 2009)	40 cycles, Annealing temperature 57 °C
		ITS5-u (Pfunder et al. 2001)/ITS4rust (Beenken et al. 2012)	35 cycles, Annealing temperature 52 °C
		Rust2inv/ITS4rust	35 cycles, Annealing temperature 55 °C
	28S (700 bp)	LRust1R (Beenken et al. 2012)/LR6 (Vilgalys and Hester 1990)	35 cycles, Annealing temperature 52 °C
		LRust1R/LRust3 (Beenken et al. 2012)	40 cycles, Annealing temperature 51 °C
Malus species	ITS1-5.8S-ITS2 (800 bp)	AB101/AB102 (Robinson et al. 2001)	40 cycles, Annealing temperature 65 °C
		ITS5/ITS4 (White et al. 1990)	40 cycles, Annealing temperature 55 °C
	rbcL gene (700 bp)	rbcLa-F (Levin 2003)/rbcLa-R (Kress and Erickson 2007)	32 cycles, Annealing temperature 55 °C
Juniperus species	ITS1-5.8S-ITS2 (800 bp)	26S-25R (Adams and Schwarzbach 2013)/ITS5 (White et al. 1990)	40 cycles, Annealing temperature 55 °C
	rbcL gene (700 bp)	rbcLa-F (Levin 2003)/rbcLa-R (Kress and Erickson 2007)	35 cycles, Annealing temperature 55 °C

Supplementary Table 8. Sequences of *Gymnosporangium* species used in the phylogenetic analyses.

Species	GenBank accession no. of rDNA ITS regions	DNA fragment	Reference*
G. amelanchieris	KM486547.1	ITS and 28S	Fernandez & Alvarado (unpublished)
	KP261040.1	ITS and 28S	Fernandez & Alvarado (unpublished)
G. asiaticum	KJ720161.1	ITS and 28S	Novick et al. (unpublished)
G. atlanticum	KP261044.1	ITS and 28S	Fernandez & Alvarado (unpublished)
	KM403108.1	ITS and 28S	Fernandez & Alvarado (unpublished)
	KM403109.1	ITS and 28S	Fernandez & Alvarado (unpublished)
G. betheli	KJ720163.1	ITS and 28S	Novick et al. (unpublished)
G. clavariiforme	KJ720164.1	ITS and 28S	Novick et al. (unpublished)
	KP261048.1	ITS and 28S	Fernandez & Alvarado (unpublished)
G. confusum	KJ720165.1	ITS and 28S	Novick et al. (unpublished)
	KP261046.1	ITS and 28S	Fernandez & Alvarado (unpublished)
	KP261042.1	ITS and 28S	Fernandez & Alvarado (unpublished)
G. connersii	KJ720166.1	ITS and 28S	Novick et al. (unpublished)
G. cupressi	KJ720169.1	ITS and 28S	Novick et al. (unpublished)
G. exiguum	KJ720170.1	ITS and 28S	Novick et al. (unpublished)
G. globosum	HQ317506.1	ITS and 28S	Liu et al. (unpublished)
	HQ317511.1	ITS and 28S	Liu et al. (unpublished)
G. gracile	KM486542.1	ITS and 28S	Fernandez & Alvarado (unpublished)
G. gracile	KM486543.1	ITS and 28S	Fernandez & Alvarado (unpublished)
G. kernianum	KJ720177.1	ITS and 28S	Novick et al. (unpublished)
G. juniperi-virginianae	DQ267127.1	ITS and 28S	Matheny et al. (unpublished)
	HQ317510.1	ITS and 28S	Liu et al. (unpublished)
	KJ720176.1	ITS and 28S	Novick et al. (unpublished)
G. kernianum	KJ720177.1	ITS and 28S	Novick et al. (unpublished)
G. miyabei	KJ720178.1	ITS and 28S	Novick et al. (unpublished)
G. multiporum	KJ720179.1	ITS and 28S	Novick et al. (unpublished)
G. nelsonii	KJ720180.1	ITS and 28S	Novick et al. (unpublished)
G. nidus-avis	KJ720181.1	ITS and 28S	Novick et al. (unpublished)
G. nipponicum	KJ720182.1	ITS and 28S	Novick et al. (unpublished)
G. sabinae	KM403110.1	ITS and 28S	Fernandez & Alvarado (unpublished)
	KP261039.1	ITS and 28S	Fernandez & Alvarado (unpublished)
	KF925317.1	ITS and 28S	Filipp & Spornberger (unpublished)
	KF925316.1	ITS and 28S	Filipp & Spornberger (unpublished)
	KF925318.1	ITS and 28S	Filipp & Spornberger (unpublished)
G. tremelloides	KJ720185.1	ITS and 28S	Novick et al. (unpublished)
G. vauqueliniae	KJ720186.1	ITS and 28S	Novick et al. (unpublished)
G. yamadae	GU058012.1	ITS and 28S	Dixon et al. (2010)
Roestelia sp.	KP308395.1	ITS and 28S	Shen et al. (unpublished)
	KP308396.1	ITS and 28S	Shen et al. (unpublished)
	KP308397.1	ITS and 28S	Shen et al. (unpublished)
Ravenelia macowaniana	KP687429.1	ITS and 28S	Ebinghaus et al. (unpublished)
R. evansii	KP687425.1	ITS and 28S	Ebinghaus et al. (unpublished)

*Reference: **Dixon LJ**, **Castlebury LA**, **Aime MC**, **Glynn NC**, **Comstock JC**. 2010. Phylogenetic relationships of sugarcane rust fungi. *Mycological Progress* **9**: 459–468.

Supplementary Table 9. GenBank accession numbers of rDNA ITS regions of *Malus* species and *Juniperus* species obtained from this study

Host plants	Specimen no[a].	Locality[b]	GenBank accession no. of rDNA ITS regions
M. asiatica	HMAS140407	China, Beijing	KU288738
M. communis	NYBG237060	USA, Kansas	KU288710
	CUP21441	USA, New York	KU288716
M. ioensis	CUP595	USA, New York	KU288709
M. malus	CUP20159	USA, New York	KU288710
	CUP20257	USA, Tennessee	KU288707
	HMAS135289	China, Yunnan	KU288740
M. micromalus	HMAS199333	China, Beijing	KU288711
	HMAS246995	China, Beijing	KU288714
	HMAS243188	China, Beijing	KU288712
	HMAS246997	China, Beijing	KU288713
M. prunifolia	HMAS36992	China, Gansu	KU288717
M. pumila	HMAS55757	China, Beijing	KU288742
	HMAS38649	China, Shaanxi	KU288743
	HMAS246994	China, Inner Mongolia	KU288725
	HMAS246996	China, Inner Mongolia	KU288726
	HMAS44394	China, Sichuan	KU288744
	HMAS14328	China, Jiangsu	KU288739
	HMAS44513	China, Hebei	KU288741
M. spectabilis	HMAS26416	China, Sichuan	KU288708
M. sylvestris	CUP56165	Finland, Aland	KU288715
J. chinensis	HMAS79065	China, Guizhou	KU288736
	HMAS79186	China, Beijing	KU288739
	HMAS82779	China, Inner Mongolia	KU288753
	HMAS8632	China, Beijing	KU288740
	HMAS165302	China, Yunnan	KU288744
	HMAS11216	China, Jiangsu	KU288738
	HMAS135611	China, Hubei	KU288744
	HMAS47228	China, Guangdong	KU288742
	HMAS172366	China, Beijing	KU288741
J. communis	HMAS2146	Scandinavia	KU288746
	HMAS24626	Canada, Ontario	KU288747
	HMAS67951	Germany, Bayem	KU288754
	NYBG23203	USA, New York	KU288748
J. horizontalis	NYBG193243	USA, California	KU288743
	NYBG33324	USA, Iowa	KU288750
J. sabina	HMAS143609	China, Guizhou	KU288727
	HMAS18554	China, Beijing	KU288761
J. virginiana	HMAS8974	China, Anhui	KU288731
	NYBG23036	USA, California	KU288730
	NYBG1391099	USA, North California	KU288735
	NYBG237085	USA, Iowa	KU288737
	NYBG237070	USA, Kansas	KU288734
	NYBG237046	China, Iowa	KU288732
	NYBG237061	USA, Kansas	KU288745
	NYBG237038	USA, Kansas	KU288752
	NYBG461220	USA, Massachusetts	KU288728
	NYBG237077	USA, New Mexico	KU288749
	NYBG237065	USA, Kansas	KU288729
	NYBG237080	Canada, Saskatchewan	KU288733

	NYBG461234	Canada, Ontario	KU288751

[a]**HMAS**: the Mycological Herbarium of Institute of Microbiology, CAS, China; **CUP**: Plant Pathology Herbarium, Cornell University, Ithaca, NY, USA; **NYBG**: New York Botanical Garden, NY, USA.

[b]Locality with name of country followed by the name of provinces or prefectures.

[c]ND: Not analyzed.

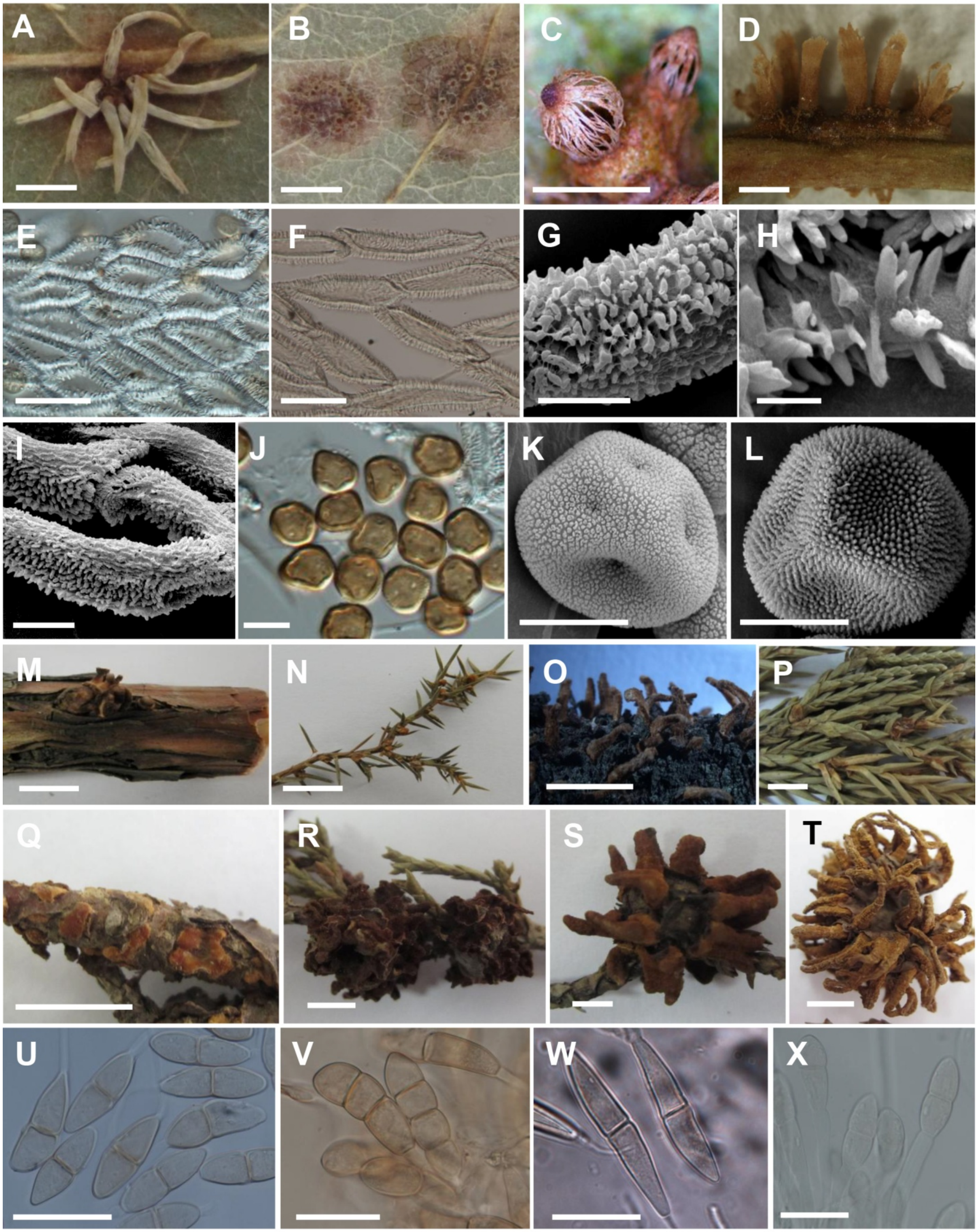

Supplementary Figure 1. Morphological variations of *Gymnopsorangium* species in aecial and telia stage. (A) roestelioid aecia. (B) aecidioid aecia. (C) peridium with cornuted apex. (D)

peridium with tubular apex. (E) oblonged or oval peridium cell. (F) rhomboidal perdium cell. (G) peridium cell verruculose with ridge-like papillae. (H) peridium cell verruculose with large papillae. (I) peridium cell verruculose. (J) globoid or ellipsoid aeciospores with 3 to 5 germ pores. (K) aeciospores with large coronate spine on the surface. (L) aeciospores with large echinulate spine on the surface. (M) fusiform and wedge-shaped telia. (N) witches' broom and hemispheric telia. (O) terete and fusiform telia. (P) witches' broom and conical telia. (Q) slight fusiform and applanate telia. (R) gall-type telia with irregular sori. (S) gall-type telia with wedge-shaped sori. (T) gall-type telia with cylindric-acuminate sori. (U) 2-celled teliospores with long and cylindrical perdicels. (V) 1 to 4 celled teliospores with long and cylindrical perdicels. (W) 2-celled teliospores narrowed above and below and with long and cylindrical perdicels. (X) 2-celled teliospores with carotiform type perdicles. Bars: A, B, C, D = 2 mm; E, F = 40 μm; G, I, K, L =10 μm; H = 2 μm; J = 20 μm; M, N, O, P, R, S = 1cm; U, V, W, X = 50 μm.

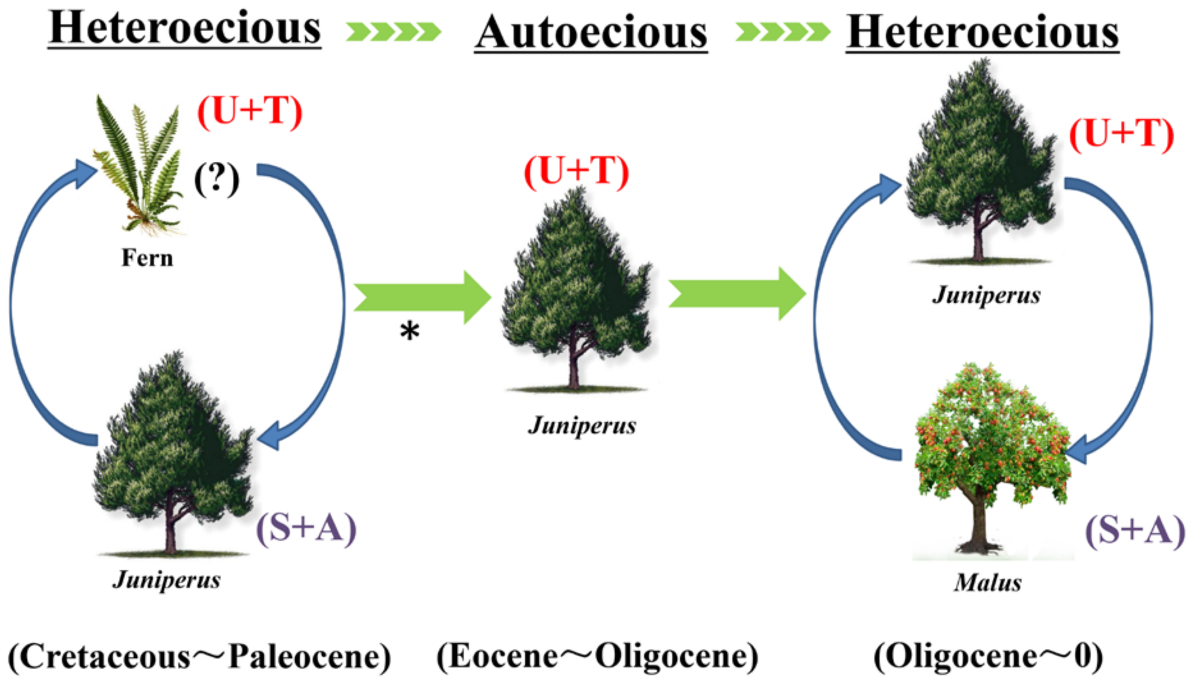

Supplementary Figure 2. Hypothesis of the evolutionary process of *Gymnosporangium* species on *Malus*. The alphabets S, A, U and T represent spermogonia, aecia, uredinia and telia in the whole life cycle, respectively. The asterisk (*) represents the reduced life cycle of the ancestor of *Gymnopsorangium* species based on the Tranzschel's Law. The question mark (?) indicates that some forest ferns were speculated as the host species of the ancestor of *Gymnosporangium* species, and uredinial and telial stages might occurred on ferns. The epochs in the parentheses indicate the estimated time period when the evolutionary pathway of rust fungi occurred.
